# Supplementary material for: Complex Formation between the Transcription Factor WRKY53 and Antioxidative Enzymes Leads to Reciprocal Inhibition
Source: Antioxidants (Basel). 2024 Mar 5;13(3):315. doi: 10.3390/antiox13030315 (PMC10967774; doi:10.3390/antiox13030315)
Supplement: Supplementary file 1 [file antioxidants-13-00315-s001.zip › Supplemental Figures S1-S4.pdf]

Complex formation between the transcription factor WRKY53 and antioxidative enzymes leads to reciprocal inhibition

Ana Gabriela Andrade Galan [1], Jasmin Doll [1], Natalie Faiß [1], Patricia Weber [1], and Ulrike Zentgraf [1,*]

[1]Center for Plant Molecular Biology (ZMBP), University of Tübingen, Auf der Morgenstelle 32, 72076 Tübingen, Germany; ana.andrade@zmbp.uni-tuebingen.de, jasmin.doll@zmbp.uni-tuebingen.de, natalie.faiss@zmbp.uni-tuebingen.de, pa.weber@student.uni-tuebingen.de, ulrike.zentgraf@zmbp.uni-tuebingen.de,
*Correspondence: ulrike.zentgraf@zmbp.uni-tuebingen.de

Supplemental Figure S1-S4

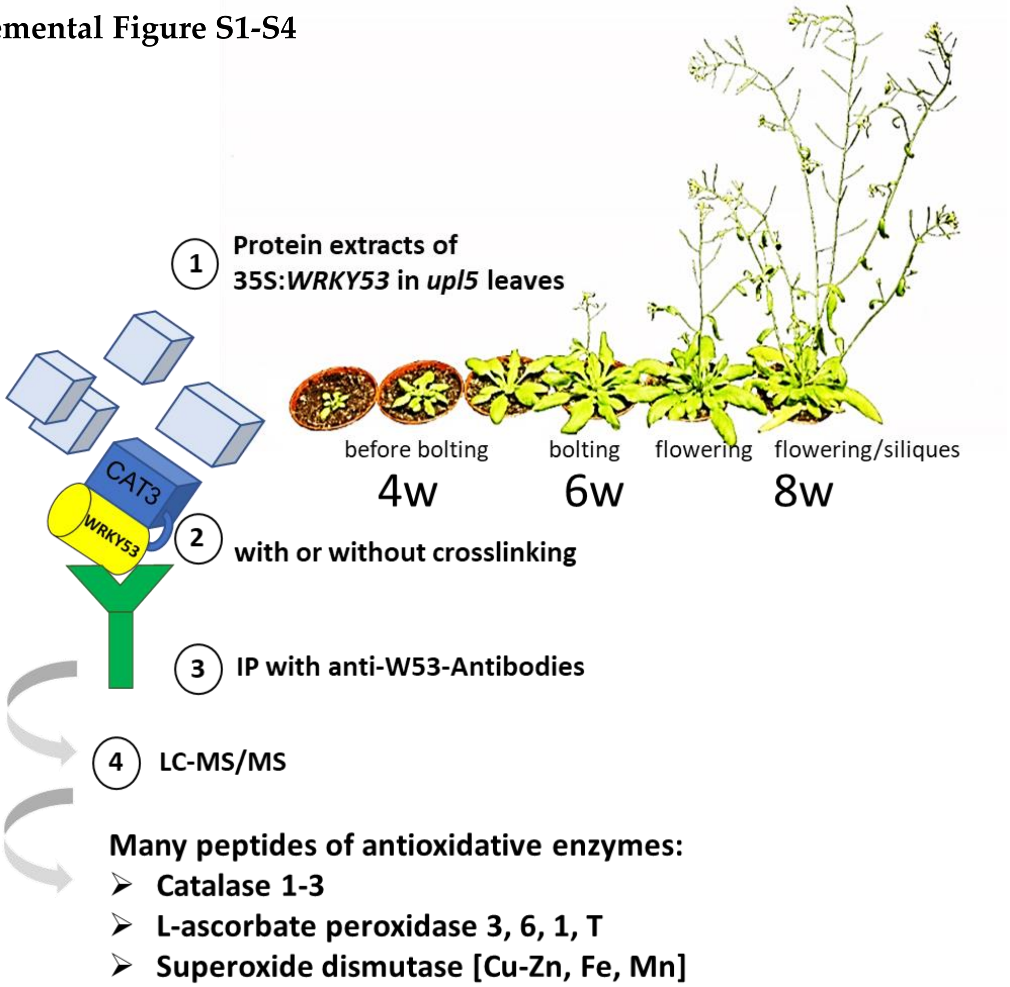

Supplemental Figure S1: Workflow to characterize *in vivo* protein interaction partners of WRKY53 using pulldown assays of WRKY53 in different developmental stages (4 week, 6 week and 8 week old plants). ① Proteins were isolated from leaves of a 35S:*WRKY53* overexpressing plants in an *upl5* mutant background. UPL5 encodes a HECT domain E3 ubiquitin ligase which is involved in the protein degradation of WRKY53, therefore higher protein levels of WRKY53 can be achieved in the *upl5* mutant background. ② Proteins were either used directly or were treated with formaldehyde or DSS for crosslinking, ③ CO-IP was performed using anti-WRKY53-Antibodies ④ Subsequently, LC-MS/MS analyses was performed with the pulled-down proteins as indicated in more detail in Supplemental Table 1, many peptides of antioxidative enzymes were identified.

Recombinant WRKY proteins

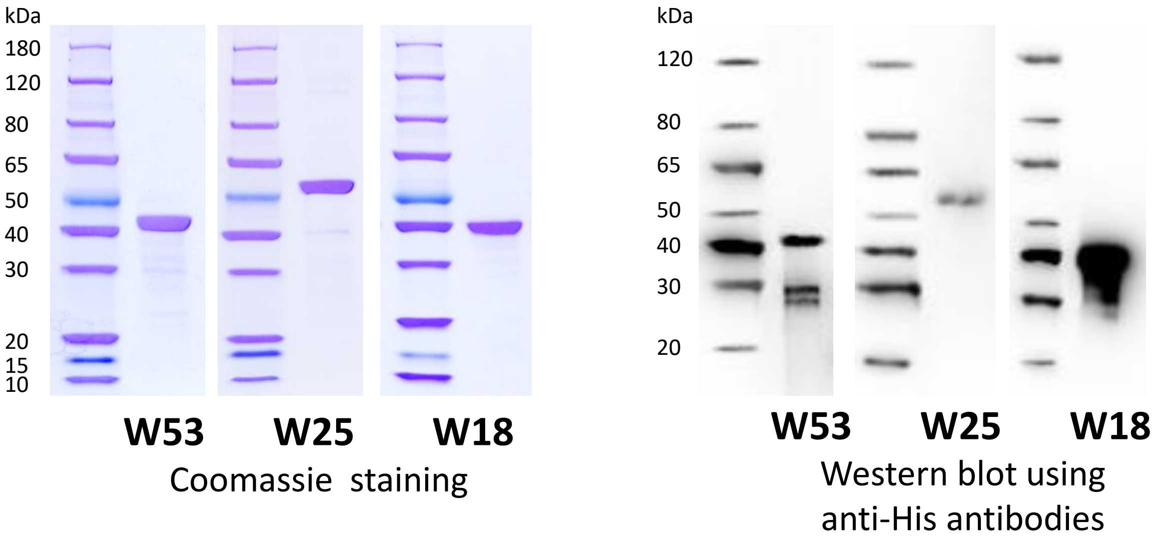

Supplemental Figure S2: SDS-PAGE of the purified proteins 8xHis tagged WRKY53, WRKY25, and WRKY18, which were used for the inhibition experiments. These proteins have been provided by Biomatik (Cambridge, Ontario, Canada).

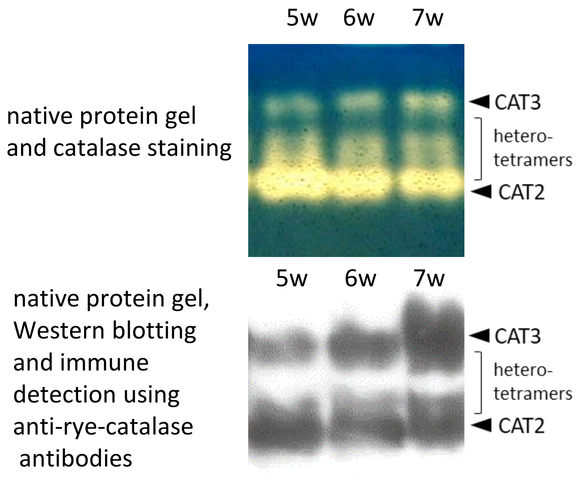

Supplemental Figure S3: Catalase activity versus protein amount.
The catalase zymogram shows catalase activities of protein extracts isolated from leaf material of 5-, 6- or 7-week-old plants (top). This native gel has been blotted and detected with anti-rye-catalase antibodies (bottom) indicating that CAT3 homotetrames are less active than CAT2 homotetrameres or heterotetrameres, if compared to the amount of proteins shown by the Wester blot.

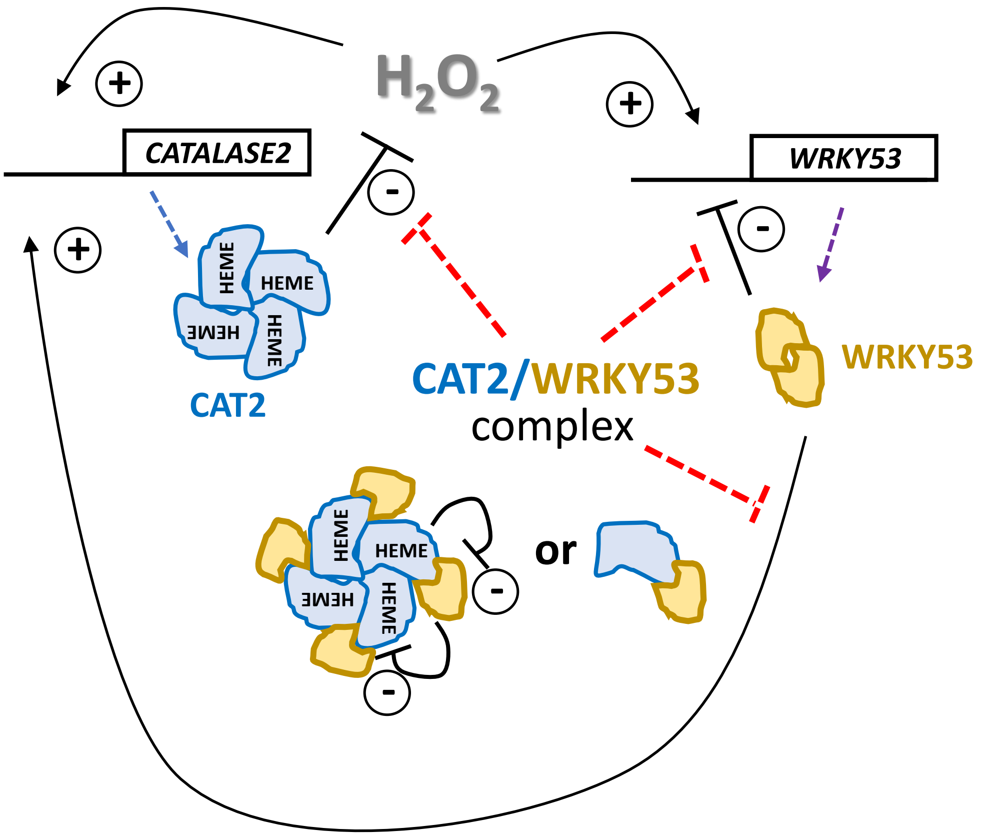

Supplemental Figure S4: Model of the feedback regulation between WRKY53 and CAT2. H_2O_2 can increase expression of the *CAT2* gene, CAT2 subunits form tetramers including a HEME group per subunit and enzyme activity can then reduce H_2O_2 content leading again to reduced gene expression (negative feed-back loop). H_2O_2 can also increase expression of the *WRKY53* gene, the WRKY53 protein can then reduce again its own expression as dimer by a direct interaction with its own promoter but also by an induction of *CAT2* gene expression which, in turn, then reduces H_2O_2 content again (negative feedback loop). However, at the same time WRKY53 protein can directly interact with either the CAT2 tetrameric complexes or only the protein subunits to inhibit CAT2 enzyme activity and WRKY53 repressor function on its own gene, both leading to increased *WRKY53* expression. This means, whenever the CAT2/WRKY53 complex is formed, WRKY53 expression is fostered, regardless whether the complex is located in the peroxisomes or in the nucleus (positive feedback loop).
